# Teriparatide Did Not Increase Adult Osteosarcoma Incidence in a 15‐Year US Postmarketing Surveillance Study

**DOI:** 10.1002/jbmr.4188

**Published:** 2020-10-13

**Authors:** Alicia Gilsenan, Kirk Midkiff, David Harris, Nicole Kellier‐Steele, David McSorley, Elizabeth B Andrews

**Affiliations:** ^1^ RTI Health Solutions Research Triangle Park NC USA; ^2^ Eli Lilly and Company Indianapolis IN USA

**Keywords:** GENERAL POPULATION STUDIES, OSTEOPOROSIS, PRIMARY TUMORS OF BONE AND CARTILAGE

## Abstract

The Osteosarcoma Surveillance Study was initiated in the United States in 2003 to monitor for a potential association between the osteoporosis treatment teriparatide and osteosarcoma. Osteosarcoma occurs at a background incidence rate of approximately 2.5 cases per million per year in US adults aged 40 years or older. For this study, incident cases of osteosarcoma diagnosed between January 1, 2003, and December 31, 2016, were identified through participating cancer registries in the United States. Information on prior exposure to medications and possible risk factors was obtained by self‐report (or proxy report) in telephone interviews. Exposure information was verified through medical record abstraction for a sample of patients. A standardized incidence ratio was estimated to compare the observed and expected numbers of osteosarcoma patients with a prior history of teriparatide treatment. Interviews were completed for 24% (1173) of patients diagnosed with osteosarcoma between 2003 and 2016; three reports of teriparatide use before diagnosis were identified. Based on the background incidence rate, the expected number of osteosarcoma cases among patients treated with teriparatide was 4.17. Given the three observed cases, the standardized incidence ratio was 0.72 (90% confidence interval [CI], 0.20 to 1.86). Demographic characteristics were similar for interviewed and noninterviewed patients. Agreement was >90% between self‐reported and chart‐recorded exposure to osteoporosis medications. Mean age of interviewed patients was 61 years; 53% of patients were male, 84% were white, and 5% were Hispanic. The prevalence of suspected risk factors for development of osteosarcoma among the osteosarcoma cohort was 19% for history of radiation and 4% for history of Paget's disease of bone. These findings showed that the incidence of osteosarcoma associated with teriparatide use during the 15‐year surveillance period was no different than would be expected based on the background incidence rate of osteosarcoma. © 2020 The Authors. *Journal of Bone and Mineral Research* published by Wiley Periodicals LLC on behalf of American Society for Bone and Mineral Research (ASBMR).

## Introduction

The 15‐year US Osteosarcoma Surveillance Study was initiated in 2003 to evaluate a potential association between the recombinant human parathyroid hormone analog teriparatide (Forteo; Eli Lilly and Company, Indianapolis, IN, USA) and osteosarcoma in humans. The study was established under a postmarketing commitment to the US Food and Drug Administration (FDA) following the November 2002 approval of teriparatide. In the United States, teriparatide is indicated for the treatment of postmenopausal women with osteoporosis at high risk for fracture, for the increase of bone mass in men with primary or hypogonadal osteoporosis at high risk for fracture, and for the treatment of men and women with osteoporosis associated with sustained systemic glucocorticoid therapy at high risk for fracture.

Preclinical evidence suggested a potential association between teriparatide and osteosarcoma. Specifically, in preclinical rat studies, a dose‐dependent increase in the risk of osteosarcoma incidence was observed after administration of teriparatide.^(^ ^1^ ^)^ However, subsequent studies demonstrated a “no‐effect” dose in rats and no bone evidence of bone tumor in a long‐term study of cynomolgus monkeys.^(^ ^2^, ^3^ ^)^ On the basis of preclinical evidence, the US prescribing information for teriparatide includes a warning about a potential risk of osteosarcoma and precautions against use of the product for patients with risk factors for osteosarcoma (eg, Paget's disease of the bone, unexplained increase in alkaline phosphatase, open epiphyses, prior radiation therapy).^(^ ^4^ ^)^ The current label recommends that lifetime treatment with teriparatide should be limited to a maximum duration of 2 years, whereas the average duration of use in patients ≥65 years has been reported to be 10 months.^(^ ^5^ ^)^

Osteosarcoma is a primary malignant bone tumor characterized by the production of osseous matrix by neoplastic cells. Little is known about the etiology of osteosarcoma in humans,^(^ ^6^, ^7^ ^)^ but potential risk factors include Paget's disease of the bone and prior radiation treatment administered to the bones.^(^ ^7^, ^8^, ^9^, ^10^ ^)^ Nevertheless, most osteosarcomas are diagnosed in patients without these risk factors.^(^ ^11^ ^)^ As estimated from National Cancer Institute Surveillance, Epidemiology, and End Results (NCI‐SEER) data, osteosarcoma occurs at a background incidence rate of approximately 2.5 cases per million per year in US adults aged ≥40 years.^(^ ^12^ ^)^

In addition to the US case‐series study, the surveillance program for teriparatide includes four other components: a companion case‐series surveillance study in Europe (completed in 2014),^(^ ^13^ ^)^ a Forteo Patient Registry in the Unites States,^(^ ^14^ ^)^ and two population‐based observational cohort studies. This US case‐series study started 90 days after the first marketed use of teriparatide and had a duration of 15 years. The objectives were to identify newly diagnosed cases of osteosarcoma among men and women aged ≥40 years and to determine incident osteosarcoma cases, if any, with a history of teriparatide treatment.

## Patients and Methods

### Study design

The methodology and interim results for this study have been described in detail previously.^(^ ^15^ ^)^ Briefly, the study used a case‐series design to identify incident cases of osteosarcoma from participating cancer registries in the US. Patients diagnosed with osteosarcoma were identified through state, regional, or comprehensive cancer treatment center cancer registries. Information on medical history and antecedent exposures, including teriparatide and other medications, was collected through a structured telephone interview with patients or their proxies. Responses were validated by medical record review in a random sample. The observed number of patients with osteosarcoma who had a confirmed exposure to teriparatide was compared with the number of exposed osteosarcoma cases expected to be identified by the cancer registries using a standardized incidence ratio (SIR) and corresponding 90% confidence interval (CI).

### Case identification

A total of 30 US cancer registries participated in the study, including population‐based registries with regional (n = 1) or state‐level catchment areas (n = 26) and hospital‐based registries affiliated with an oncology referral center (n = 3). Participating registries were asked to use prespecified International Classification of Diseases for Oncology, 3rd edition (ICD‐O‐3; Geneva, Switzerland: WHO) codes to identify patients with osteosarcoma during the study period (April 2004–October 2018). Registries collect pertinent tumor‐related information (eg, date of diagnosis, morphology, topography) as part of their standard case‐ascertainment process, and they capture diagnoses from both inpatient and outpatient treatment facilities. Because reporting of incident cancer cases to cancer registries is legally mandated, these registries were ideal for identifying a cancer population.

Eligible patients had been diagnosed with osteosarcoma on or after January 1, 2003, and had a primary residence in the United States. The case definition for study inclusion was meeting all of the following criteria: (i) patient aged ≥40 years at the time of diagnosis; (ii) diagnosis reported from a participating cancer registry; (iii) diagnosis of osteosarcoma based on at least one of 12 ICD‐O‐3 oncology codes for osteosarcoma or one of five other ICD‐O‐3 oncology codes for which the primary tumor site was bone.

Cases were obtained from participating cancer registries, which provided the date of diagnosis, the primary tumor site, and the tumor morphology according to 12 ICD‐O‐3 morphology codes meeting the definition of osteosarcoma, with no restriction on the primary site of the tumor (Table 1). In addition, to capture cases of osteosarcoma possibly misclassified as another similar cancer,^(^ ^7^ ^)^ additional cancers were included if diagnosed with one of five ICD‐O‐3 morphology codes with a primary tumor site listed as bone (Table 1). Patient characteristics, medication exposure, and history and potential risk factors for patients diagnosed with these additional five ICD‐O‐3 morphology codes are presented in the Supplementary Appendix S1.

**Table 1:** Definition of Osteosarcoma Diagnosis

ICD‐O‐3 morphology codes^a^	Additional ICD‐O‐3 codes^b^
9180/3 Osteosarcoma NOS 9181/3 Chondroblastic osteosarcoma 9182/3 Fibroblastic osteosarcoma 9183/3 Telangiectatic osteosarcoma 9184/3 Osteosarcoma in Paget's disease of bone 9185/3 Small cell osteosarcoma 9186/3 Central osteosarcoma 9187/3 Intraosseous well‐differentiated osteosarcoma 9192/3 Parosteal osteosarcoma 9193/3 Periosteal osteosarcoma 9194/3 High‐grade surface osteosarcoma 9195/3 Intracortical osteosarcoma	8800/3 Sarcoma, NOS 8801/3 Spindle cell sarcoma 8810/3 Fibrosarcoma, NOS 8830/3 Malignant fibrous histiocytoma 9243/3 Dedifferentiated chondrosarcoma

ICD‐O‐3 = International Classification of Diseases for Oncology, 3rd edition; NOS = not otherwise specified.

^a^ These 12 ICD‐O‐3 morphology codes, with no restriction on the primary site of the tumor, met the definition of osteosarcoma.

^b^ Similar cancers were defined as cases diagnosed with one of five additional ICD‐O‐3 morphology codes and were included in the study if the primary tumor site was listed as bone (but were analyzed separately from cases identified via the ICD‐O‐3 osteosarcoma codes).

### Data collection

Patients identified as having osteosarcoma were contacted regarding participation in telephone interviews; procedures were customized to the requirements of each registry and have been described in detail.^(^ ^15^, ^16^ ^)^ Briefly, registries provided patient contact details and information about cancer diagnosis information to the study investigators. Investigators then contacted the patient or proxy, having obtained approval from the physician listed in the registry record as necessary.

During 30‐min telephone interviews with computer assistance, trained interviewers collected information about patients' treatment with teriparatide, potential risk factors for osteosarcoma (lifestyle exposures; treatment, injury, and infection history; environmental exposures; and personal and family health history), and demographics. If the patient was deceased or unable to participate in the interview, a proxy familiar with the patient's medical history was interviewed. Capture of self‐reported exposure to teriparatide or other similar medications (ie, those stored in the refrigerator and administered by daily injection) was facilitated using a structured, deductive question series. All instances of self‐reported teriparatide treatment or daily injections for any reason underwent review and adjudication to investigate possible exposure to teriparatide. Data collection was initiated in July 2004 for patients diagnosed on or after January 1, 2003. Beginning in September 2008, in an effort to improve the response rate for the telephone interviews, patients or proxies participating in the interviews received $25 compensation for their time.

Self‐reported information provided during the telephone interview was validated among a randomly sampled subset of patients to assess agreement between some self‐reported telephone interview responses by patient or proxy and abstracted medical record data. A minimum of 10% of all patients interviewed or 25 patients annually (whichever was greater) were selected for validation. Eligible patients must have returned a Health Insurance Portability and Accountability Act (HIPAA) release form and listed a primary care physician seen before the qualifying cancer diagnosis. Trained medical record abstractors contacted the treating physician, obtained the records, and abstracted the information. The abstractors provided the completed abstraction forms for data entry and analysis of the level of agreement between self‐reported information and the medical record.

### Statistical analyses

Cancer case counts were summarized separately for patients with an ICD‐O‐3 code indicating osteosarcoma and for patients diagnosed with one of the five additional ICD‐O‐3 codes for which the primary tumor site was bone. Descriptive analyses were conducted to summarize osteoporosis history and treatments, including teriparatide, demographics, medical and family history, and lifestyle and environmental exposures. Prior teriparatide exposure was derived from interview data. See Andrews and colleagues^(^ ^15^ ^)^ for additional details on the analyses.

The observed number of patients with osteosarcoma and a confirmed exposure to teriparatide was compared with the number of exposed osteosarcoma cases expected to be identified by the cancer registries using a SIR. The expected number of osteosarcoma cases among patients treated with teriparatide was estimated based on the background rate of osteosarcoma in the United States (ie, assuming no association between drug exposure and disease), age‐ and sex‐adjusted to the teriparatide‐treated population, and the estimated number of cumulative person‐years at risk among patients treated with teriparatide in the United States. The estimate was further refined to account for the osteosarcoma cases that the study identified and the number of patients interviewed. The analysis years (2003–2016) were restricted to diagnosis years, with more complete case ascertainment and reporting from participating cancer registries, and excluded diagnosis year 2017. Precision of the SIR estimator was evaluated using exact 90% CIs around the ratio, produced by the following formulas:

(1) $${\mathrm{SIR}}_{\text{lower bound}}=\chi_{0.05,2D}^{2}/2E$$

(2) $${\mathrm{SIR}}_{\text{upper bound}}=\chi_{0.95,2\left(D+1\right)}^{2}/2E$$

where *D* = observed number patients with osteosarcoma reporting teriparatide use; *E* = the expected number of osteosarcoma cases among teriparatide users; $\chi_{\alpha ,\upsilon }^{2}$ = the α percentile of the chi‐square distribution with *υ* degrees of freedom, with α = 0.05 and *υ* = 2*D* for the *SIR* lower bound, and α = 0.95 and *υ* = 2(*D* + 1) for the *SIR* upper bound.

Sensitivity analyses were conducted to examine changes in the SIR by varying the observed and expected number of osteosarcoma cases among teriparatide users. The observed number of cases was increased by including the five additional ICD‐O‐3 codes (8800, 8801, 8810, 8830, 9243) for which the primary site was bone. The expected number of osteosarcoma cases among teriparatide users was recalculated for a variety of plausible alternative values for the data components from which the number was derived: (i) the background incidence rate of osteosarcoma, (ii) the estimated person‐years at risk, and (iii) the estimated interview rate obtained in the surveillance study.

### Ethics approval

The study was approved by the RTI International institutional review board and local institutional review boards and other committees affiliated with the cancer registries.

## Results

### Participant characteristics

**Fig 1:**
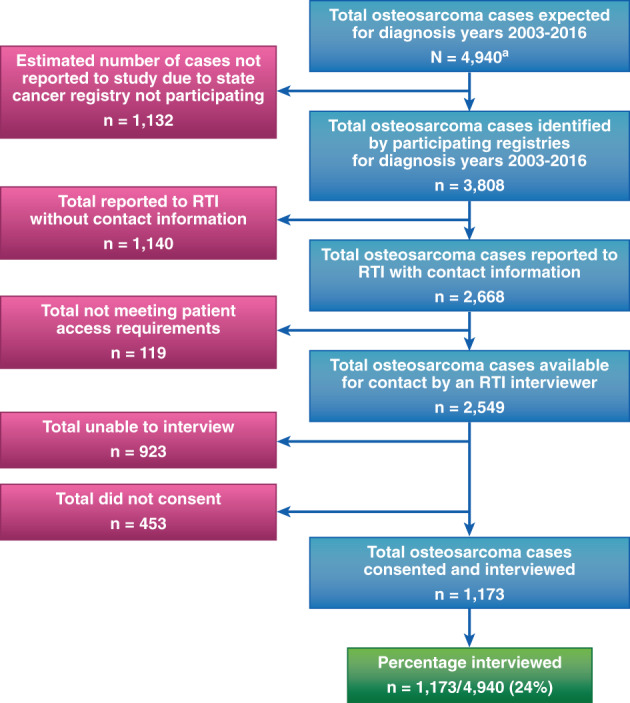
Osteosarcoma Surveillance Study progress, diagnosis years 2003 to 2016, as of December 31, 2018. ^a^Diagram excludes patients diagnosed in 2017 due to a 9‐month to 18‐month lag between diagnosis and reporting from participating registries to RTI. Estimated using the SEER rate of osteosarcoma, 2.5 per million population per year,^(^ ^12^ ^)^ applied to “Annual Estimates of the Resident Population by Age and Sex for States” from 2003 to 2016.^(^ ^19^ ^)^ NCI = National Cancer Institute; RTI = RTI International; SEER = Surveillance, Epidemiology, and End Results.

A total of 3808 patients diagnosed with osteosarcoma from 2003 through 2016 were identified by the 30 cancer registries that contributed data during the study; 4940 patients were expected in the United States for that period, thus 77% of the expected osteosarcoma patients were identified by the participating registries. Of the 3808 patients identified by the registries, 2549 were reported to the study center and met all necessary requirements to be contacted for an interview. A total of 1173 (31%) patients or their proxies were interviewed; therefore, based on the expected total of all osteosarcoma cases (4940 expected), 24% of patients or their proxies were interviewed (Fig. 1).

Among the 1173 patients with osteosarcoma who were interviewed or whose proxies were interviewed, more than one‐half were men (53%), most were white (84%), and 5% were Hispanic. The mean age at the time of diagnosis of osteosarcoma was 61 years (range, 40–94 years). More than one‐half of patients (918/1173) were living when their osteosarcoma was reported (Table 2). The most common osteosarcoma ICD‐O‐3 morphology codes were for osteosarcoma not otherwise specified (*n* = 832; 71%), chondroblastic osteosarcoma (*n* = 148; 13%), and fibroblastic osteosarcoma (*n* = 77; 7%). The anatomical site of the tumor varied, but the lower extremities predominated, with nearly one‐half of cases occurring in the legs (33%) or the pelvic region (14%). The next most prevalent sites included the skull or face region (16%), connective or soft tissue (10%), and the upper limbs (9%).

**Table 2:** Demographic and Disease Characteristics

Characteristic	Total identified by registries (*n* = 3808)	Interviewed cases (*n* = 1173)
Age at diagnosis (years), mean ± SD, range	62.6 ± 13.4; 40–99	61.2 ± 12.7; 40–94
Sex, *n* (%)		
Female	1863 (49)	556 (47)
Male	1945 (51)	617 (53)
Hispanic origin, *n* (%)		
No	3081 (81)	926 (79)
Yes	316 (8)	56 (5)
Unknown	411 (11)	191 (16)
Race, *n* (%)		
Black	436 (11)	118 (10)
White	2852 (75)	989 (84)
Other	161 (4)	34 (3)
Unknown	359 (9)	32 (3)
Vital status, *n* (%)		
Deceased	1617 (42)	249 (21)
Living	2159 (57)	918 (78)
Unknown	32 (1)	6 (1)
ICD‐O‐3 code, *n* (%)		
9180 Osteosarcoma NOS	2656 (70)	832 (71)
9181 Chondroblastic osteosarcoma	455 (12)	148 (13)
9182 Fibroblastic osteosarcoma	257 (7)	77 (7)
9183 Telangiectatic osteosarcoma	77 (2)	21 (2)
9184 Osteosarcoma in Paget's disease of bone	104 (3)	16 (1)
9185 Small cell osteosarcoma	25 (1)	8 (1)
9186 Central osteosarcoma	83 (2)	25 (2)
9187 Intraosseous well differentiated osteosarcoma	10 (<1)	2 (<1)
9192 Parosteal osteosarcoma	103 (3)	34 (3)
9193 Periosteal osteosarcoma	22 (1)	7 (1)
9194 High‐grade surface osteosarcoma	16 (<1)	3 (<1)
Cancer site category, *n* (%)		
Leg bones	1,128 (30)	386 (33)
Pelvis/sacrum/coccyx	596 (16)	164 (14)
Skull/face/mandible	578 (15)	182 (16)
Connective and soft tissue	369 (10)	119 (10)
Scapula/hand/arm bones	308 (8)	100 (9)
Bone and joints (unspecified)	231 (6)	52 (4)
Ribs/sternum/clavicle	206 (5)	75 (6)
Vertebrae	160 (4)	40 (3)
Breast	80 (2)	19 (2)
Other	136 (4)	32 (3)
Unknown	16 (<1)	4 (<1)

ICD‐O‐3 = International Classification of Diseases for Oncology, 3rd edition; NOS = not otherwise specified.

### Medication exposure

Among the interviews completed for patients diagnosed with osteosarcoma, 144 respondents (12%) reported history of osteoporosis and 12 (1%) reported possible prior use of teriparatide. After additional follow‐up (with patient, caregiver, and/or provider) to confirm exposure to teriparatide, three exposures were considered valid (two female, one male), eight were confirmed to be incorrect (because of patient or proxy confusion with the name of the medication), and the remaining one patient reported teriparatide use that was confirmed by their healthcare provider to have been prescribed after osteosarcoma diagnosis. Of the three exposures considered valid, one was observed approximately 8 years after starting teriparatide, another approximately 3 months after initial exposure, and the third approximately 2 years after starting teriparatide. All had diagnosis of ICD‐O3 histology code “Osteosarcoma, NOS” and the site of tumor was different for each case.

### Patient history and potential risk factors

Among all patients diagnosed with osteosarcoma who had completed interviews, 181 patients (15%) had a prior injury or infection in the same bone as the site of their bone cancer. More than one‐quarter of patients (314/1173; 27%) reported history of another type of cancer before the osteosarcoma diagnosis, 226 (19%) reported history of X‐ray or radiation treatment for prior conditions, and 127 (11%) reported history of chemotherapy for prior conditions. Paget's disease of the bone was reported by 46 patients (4%). Of patients who had radiation therapy before developing osteosarcoma, 37% (*n* = 83) developed osteosarcoma in the exact site of the radiation therapy, and an additional 36% (*n* = 82) developed osteosarcoma in the same region of the body as the radiation therapy. The most commonly reported cancers in patient family histories were breast cancer (23%), brain cancer (8%), and leukemia (6%). Only 52 patients (4%) reported an immediate blood relative with prior osteosarcoma that started in the bone.

Nearly one‐half of patients (49%) reported that they had smoked at least 100 cigarettes in their lifetime, and the majority of patients (63%) consumed alcohol in the year before osteosarcoma diagnosis. Among the environmental exposures assessed over each patient's lifetime, pesticides were the most prevalent given farm proximity (24%) or manufacturing (5%). Petrochemicals (12%) and nuclear facility proximity (7%) were less common, and only 80 patients (7%) reported prior occupations with radiation exposure (eg, X‐ray technician, dental hygienist, radiology technician).

### SIRs

#### Primary analysis

Among patients treated from 2003 through the end of 2016, 5,432,764 person‐years of risk were estimated. A background incidence rate of osteosarcoma of 3.2 cases per million per year—derived from the NCI‐SEER rate (2.5 per million population per year) and age‐ and sex‐adjusted to the teriparatide‐treated population—and study interview rate of 24% were estimated. Given these parameters, the expected number of cases of osteosarcoma exposed to teriparatide treatment is 4.17 cases. With three observed cases, the SIR is 0.72 (90% CI, 0.20–1.86).

#### Sensitivity analyses

The following alternative values of the components for the expected numbers of osteosarcoma cases were considered in five scenarios for the sensitivity analyses of the SIR. First, along with including five additional ICD‐O‐3 codes, induction periods of 1, 2, and 3 years were included. Second, a lower background incidence rate of osteosarcoma of 2.5 cases per million per year was assumed (with and without the five additional ICD‐O‐3 codes). Third, the estimated person‐years at risk were decreased by 25% (with and without the five additional ICD‐O‐3 codes). Fourth, a lower estimated interview rate in the study—20% of cases instead of 24%—was assumed (with and without the five additional ICD‐O‐3 codes). Fifth, a combination of the aforementioned values was assumed that included a decrease in the estimated person‐years at risk by 25%, an interview rate of 20% of cases (with and without the five additional ICD‐O‐3 codes), and background incidence of 2.5 cases per million per year (with the five additional ICD‐O‐3 codes).

From the recalculated observed and expected numbers of osteosarcoma cases, a series of SIRs (with CIs) for the alternative values of the data components was generated. Table 3 displays the observed and the expected numbers for each exploratory scenario. Among all scenarios explored, none produced a 90% lower confidence bound that exceeded 1.0.

**Table 3:** Standardized Incidence Ratio Sensitivity Analyses

	Number of cases			
Analysis	Observed	Expected^a^	SIR	90% CI
Based on the reference values^b^	3	4.17	0.72	0.20–1.86
Include 5 other ICD‐O‐3 codes (8800, 8801, 8810, 8830, 9243)				
1 additional case (chondrosarcoma) gets included	4	4.17	0.96	0.33–2.19
Include an induction period with 5 other ICD‐O‐3 codes (8800, 8801, 8810, 8830, 9243)				
1 year (1 case gets excluded and person‐years decreases to 4,651,698)	3	3.57	0.84	0.23–2.17
2 years (2 cases get excluded and person‐years decreases to 3,934,149)	2	3.02	0.66	0.12–2.08
3 years (3 cases get excluded and person‐years decreases to 3,282,047)	1	2.52	0.40	0.02–1.88
Assume a background rate of 2.5 per million per year	3	3.26	0.92	0.25–2.38
Include 5 other ICD‐O‐3 codes (8800, 8801, 8810, 8830, 9243)	4	3.26	1.23	0.42–2.81
Decrease person‐years by 25% (4,074,573)	3	3.13	0.96	0.26–2.48
Include 5 other ICD‐O‐3 codes (8800, 8801, 8810, 8830, 9243)	4	3.13	1.28	0.44–2.93
Assume a lower estimated study coverage of 20%	3	3.48	0.86	0.24–2.23
Include 5 other ICD‐O‐3 codes (8800, 8801, 8810, 8830, 9243)	4	3.48	1.15	0.39–2.63
In combination (decrease person‐years by 25%, 20% study coverage)	3	2.61	1.15	0.31–2.97
Include 5 other ICD‐O‐3 codes (8800, 8801, 8810, 8830, 9243)	4	2.61	1.53	0.52–3.51
Assume a background rate of 2.5 per million per year	4	2.04	1.96	0.67–4.49

CI = confidence interval; ICD‐O‐3 = International Classification of Diseases for Oncology, 3rd Edition; SEER = Surveillance, Epidemiology, and End Results; SIR = standardized incidence ratio.

^a^ Expected = person‐years at risk × background rate × study coverage.

^b^ Reference values: 5,432,764 is the estimated age‐ and mortality‐adjusted person‐years at risk following exposure to teriparatide; 3.2 cases per million per year was derived from the incidence rate from the National Cancer Institute SEER program, age‐ and sex‐adjusted to the teriparatide‐treated population; 24% is the study coverage, which is the number of patients with osteosarcoma interviewed to determine if the patient had taken teriparatide, divided by the estimated total number of patients aged 40 years and older who diagnosed with osteosarcoma in the United States for diagnosis years 2003–2016.

### Validation

For the validation component of the study, 1009 patients (or their proxies) who completed the interview and signed and returned a medical release form were considered “selection eligible” for the medical record abstraction component of the study. A random sample of 482 cases was selected; in addition, eight of the 13 cases for which possible teriparatide use was reported during the interview (12 cases for patients diagnosed with osteosarcoma and one case for patients diagnosed with one of the five additional ICD‐O‐3 codes) were included in the validation sample (five were excluded for not having returned the release for medical record abstraction). Of these 490 selected cases, medical records were abstracted for 351 patients. For 139 patients, records could not be obtained or contained insufficient information.

Agreement was high between answers provided during the telephone interview and data abstracted from the patient's medical records: 96% or higher agreement for osteoporosis medication use (except for alendronate [Fosamax; Merck and Co., Kenilworth, NJ, USA], which was 92%), 85% agreement for history of osteoporosis, 89% or higher agreement for history of radiation or chemotherapy treatment or history of prior cancer, and 97% agreement for history of Paget's disease.

### Generalizability

Among patients with osteosarcoma, there were no notable differences in the distributions for age at diagnosis (age categories, mean age, and range), sex, or ethnicity between all patients identified by participating registries and patients interviewed (see Table 2). White individuals were slightly overrepresented among the patients interviewed (84%) compared with all patients identified (75%). As anticipated, a higher proportion of interviews were completed for patients reported by the registries to be alive at the time of diagnosis (78%) than for the total number of eligible patients identified by the registries and reported as alive (57%). Conversely, the proportion of interviews completed by proxies for patients reported by the registries to be deceased (21%) was lower than the total number of eligible patients identified by the registries and reported to be deceased (42%). The distribution of ICD‐O‐3 morphology codes and primary tumor sites was similar between identified and interviewed patients.

## Discussion

At study conclusion, 24% of incident cases of osteosarcoma diagnosed in the United States from 2003 through 2016 among men and women aged 40 years or older had completed interviews. Three patients had valid teriparatide exposure before diagnosis of osteosarcoma (primary case group diagnosed with one of the 12 ICD‐O‐3 codes); an additional case was identified in the similar case group defined by the five additional ICD‐O‐3 codes. The histological type for all three cases was the most common overall (Osteosarcoma, NOS) and location of the tumor varied for each exposed case. Although 53% of osteosarcoma cases identified were male, two out of three exposed cases were female. This is not unexpected given that approximately 90% of Forteo users are female (data on file).

The SIR for the three observed cases in the osteosarcoma case group was 0.72 (90% CI, 0.20–1.86), and the lower bound of the 90% CI for the SIR did not exceed the null value of 1.0. Even with inclusion of the additional case in the SIR calculation from the patients identified by diagnosis with one of the five additional ICD‐O‐3 codes, the lower bound of the SIR did not exceed 1. Therefore, diagnostic misclassification is unlikely to explain the lack of an association observed in this study. In a post hoc subgroup analysis among females, the SIR was 0.61 (90% CI, 0.11–1.93). For statistical evidence of an association between teriparatide treatment and osteosarcoma, the lower bound of the 90% CI for the SIR would need to exceed 1.0. Nine cases reporting prior teriparatide use would need to have been observed during the same period for the lower bound of the 90% CI to exceed 1.0. The study had statistical power to detect a potential threefold increased risk, had this existed, or one additional case of osteosarcoma per 156,000 teriparatide‐treated patients per year.

This long‐term case‐series study of adult patients aged ≥40 years with osteosarcoma provides additional information relating to the demographics, tumor characteristics, and potential risk factors for a large, population‐based sample of patients with a rare disease. The most common morphology was osteosarcoma not otherwise specified, and the most common tumor site was the lower extremities, consistent with clinical expectations and the European component of this study. In addition, approximately 19% of patients interviewed reported having received radiation therapy before developing osteosarcoma; of these, 37% developed osteosarcoma in the exact site of the radiation therapy and an additional 36% developed osteosarcoma in the same region of the body as the radiation therapy. Patients diagnosed with osteosarcoma and interviewed in this study reflected the age, sex, and ethnicity of all patients identified by participating registries. In addition, tumor type and site were similar. White patients were slightly overrepresented in the interviewed population. These results can be generalized to the US population.

Other noninterventional studies have examined the potential relationship between teriparatide and osteosarcoma as part of an osteosarcoma surveillance program; findings from those studies to date are consistent with findings from this study. These studies include a 10‐year case‐finding surveillance study conducted in the five Nordic countries that identified 129 cases of osteosarcoma, for which 112 patient medical records were abstracted (European Network of Centres for Pharmacoepidemiology and Pharmacovigilance [ENCePP] identifier EUPAS8540; http://www.encepp.eu/encepp/viewResource.htm;jsessionid?id=8541). None of these 112 patients had a record of teriparatide use. However, given the small study size, identification of a teriparatide‐exposed patient would have occurred only if teriparatide were associated with a large increased risk of osteosarcoma.^(^ ^17^ ^)^ Two other, US‐based studies included a population‐based comparative study linking pharmacy claims data from Medicare with cancer registry data from 26 states and a population‐based comparative study linking pharmacy claims from a large commercial data source with cancer registry data from 29 states.^(^ ^18^ ^)^ Analyses from these studies were recently completed, and results are forthcoming. Finally, in a US study, the Forteo Patient Registry, as of the final annual linkage of data from more than 75,000 registered teriparatide‐exposed patients with 42 state cancer registries, no incident cases of osteosarcoma have been identified (data on file).

Some limitations of this study must be considered. First, given the case‐series study design, no comparison group was available to evaluate potential risk factors (eg, history of bone fractures, joint replacement, infection or trauma at the tumor site). Interviewing all identified patients was not feasible, and there was heterogeneity in access to patients among registries because of different state and institutional‐level research approval requirements and different mechanisms in place to protect patient privacy. It is possible that these factors limited generalizability and increased possible selection bias; however, the study cohort was evaluated, and interviewed patients were found to be generally similar to all eligible patients identified by participating registries with respect to demographic and tumor‐specific characteristics. Residual information bias may have resulted from exposure misclassification. This bias may be caused by inaccurate recall by patients or inaccuracies as a result of the time elapsed from diagnosis to study interview or from reliance on proxy interviews—38% of interviewees were adult proxies. However, misclassification of teriparatide exposure was expected to be limited owing to its unique drug delivery system (daily injections) and storage requirements (refrigeration). In addition, validation using medical record data demonstrated high agreement (>85%) between interview responses and patient records. Although it is well known that induction and latency periods exist between exposure to a carcinogen and development of cancer, the expected number of osteosarcoma cases among exposed patients was determined assuming no induction period. However, assuming longer periods of latency up to 3 years did not affect the interpretation of the study findings and only one case was diagnosed >3 years after starting teriparatide (~8 years), so considering a longer induction period would serve only to further lower the SIR (eg, an 8‐year induction period would yield an SIR of 0). Furthermore, uncertainty may exist regarding estimated expected values that were not accounted for in CI calculations. Finally, the measure obtained from this analysis is limited to the age‐ and sex‐adjusted SIR, which may suffer from residual confounding because of differences other than age and sex between patients exposed to teriparatide and the US cancer registry population. Given approximately 90% of teriparatide users are female whereas approximately 53% of osteosarcoma cases identified by the registries were male, the study cohort may not be representative of the population treated with teriparatide in terms of sex distribution.

In conclusion, this study found that the incidence of osteosarcoma associated with teriparatide use during the 15‐year surveillance period was no different than would be expected based on the background incidence rate of osteosarcoma. Three patients diagnosed with osteosarcoma reported prior teriparatide exposure; this count was below the estimated expected given estimated inputs for person‐years of risk since drug launch, background osteosarcoma incidence rates, and study interview rate. The study had the statistical ability to detect a threefold increase in risk, which, for an outcome so rare, would result in one additional osteosarcoma diagnosis per 156,000 teriparatide‐treated patients per year.

## Disclosures

This study was performed under a research contract between RTI Health Solutions and Eli Lilly and Company and was funded by Eli Lilly and Company. Elizabeth Andrews, Alicia Gilsenan, David Harris, David McSorley, and Kirk Midkiff are or were salaried employees of RTI Health Solutions. Nicole Kellier‐Steele is a salaried employee of Eli Lilly and Company. The contract between RTI Health Solutions and Eli Lilly and Company assures independent publication rights for RTI Health Solutions. The authors have no other conflict of interest to declare.

## Supporting information


**Supplemental Appendix** Results for Patients With Other Diagnostic Codes

## References

[1] Vahle JL , Sato M , Long GG , et al. Skeletal changes in rats given daily subcutaneous injections of recombinant human parathyroid hormone (1‐34) for 2 years and relevance to human safety. Toxicol Pathol. 2002;30(3):312–21. 12051548 10.1080/01926230252929882

[2] Vahle JL , Long GG , Sandusky G , Westmore M , Ma YL , Sato M . Bone neoplasms in F344 rats given teriparatide [rhPTH(1‐34)] are dependent on duration of treatment and dose. Toxicol Pathol. 2004;32(4):426–38. 15204966 10.1080/01926230490462138

[3] Vahle JL , Zuehlke U , Schmidt A , Westmore M , Chen P , Sato M . Lack of bone neoplasms and persistence of bone efficacy in cynomolgus macaques after long‐term treatment with teriparatide [rhPTH(1‐34)]. J Bone Miner Res. 2008;23(12):2033–9. 18684088 10.1359/jbmr.080807

[4] Eli Lilly and Company . Forteo [package insert]. Indianapolis, IN, USA: Eli Lilly and Company; 2002. Revised 2012.

[5] Gilsenan A , Midkiff, K. , Harris, D. , McQuay, L. , Hunter, S. , Kellier‐Steele, N. , Andrews, E. Assessing the incidence of osteosarcoma among teriparatide users via linkage of data from Medicare Part D and multiple state cancer registries in the United States. [Presented at the 35th International Conference on Pharmacoepidemiology & Therapeutic Risk Management; August 24‐28 2019. Philadelpha, PA].

[6] Fletcher CDM , Unni K , Mertens F , eds. Pathology and genetics: tumours of soft tissue and bone. Lyon, France: IARC Press; 2002. (WHO Classification of Tumours, 3rd ed., vol. 5).

[7] Unni KK , Dahlin DC . Dahlin's bone tumor: general aspects and data on 11,087 cases. 5th ed. Philadelphia: Lippincott‐Raven; 1996.

[8] Grimer RJ , Cannon SR , Taminiau AM , et al. Osteosarcoma over the age of forty. Eur J Cancer. 2003;39(2):157–63. 12509946 10.1016/s0959-8049(02)00478-1

[9] Mirabello L , Troisi RJ , Savage SA . International osteosarcoma incidence patterns in children and adolescents, middle ages and elderly persons. Int J Cancer. 2009;125(1):229–34. 19330840 10.1002/ijc.24320 PMC3048853

[10] Mirabello L , Troisi RJ , Savage SA . Osteosarcoma incidence and survival rates from 1973 to 2004: data from the Surveillance, Epidemiology, and End Results program. Cancer. 2009;115(7):1531–43. 19197972 10.1002/cncr.24121 PMC2813207

[11] Savage SA , Mirabello L . Using epidemiology and genomics to understand osteosarcoma etiology. Sarcoma. 2011;2011:548151. 21437228 10.1155/2011/548151 PMC3061299

[12] National Cancer Institute (NCI) . SEER Program. SEER*Stat Database: Incidence ‐ SEER 18 Regs Research Data + Hurricane Katrina Impacted Louisiana Cases, Nov 2012 Sub (2000‐2010) <Katrina/Rita Population Adjustment> ‐ Linked To County Attributes ‐ Total U.S., 1969‐2011 Counties, National Cancer Institute, DCCPS, Surveillance Research Program, Surveillance Systems Branch, released April 2013, based on the November 2012 submission. Bethesda, MD: NCI; 2013. Available from: https://seer.cancer.gov/seerstat/. Accessed March 14, 2019.

[13] von Schéele B , Martin RD , Gilsenan AW , et al. European postmarketing adult osteosarcoma surveillance study: characteristics of patients: a preliminary report. Acta Orthop. 2009;80(Suppl 334):67–74.19234888

[14] Gilsenan A , Harding A , Kellier‐Steele N , Harris D , Midkiff K , Andrews E . The Forteo patient registry linkage to multiple state cancer registries: study design and results from the first 8 years. Osteoporos Int. 2018;29(10):2335–43. 29978254 10.1007/s00198-018-4604-8 PMC6154045

[15] Andrews EB , Gilsenan AW , Midkiff K , et al. The US postmarketing surveillance study of adult osteosarcoma and teriparatide: study design and findings from the first 7 years. J Bone Miner Res. 2012;27(12):2429–37. 22991313 10.1002/jbmr.1768 PMC3546381

[16] Midkiff KD , Andrews EB , Gilsenan AW , et al. The experience of accommodating privacy restrictions during implementation of a large‐scale surveillance study of an osteoporosis medication. Pharmacoepidemiol Drug Saf. 2016;25(8):960–8. 27091234 10.1002/pds.4008 PMC5074316

[17] Midkiff K. Forteo/Forsteo post‐approval osteosarcoma surveillance study (Study B3D‐MC‐GHBX). Research Triangle Park, NC, USA: RTI Health Solutions; 2014 [cited 2020 Oct 1]. Available from: http://www.encepp.eu/encepp/openAttachment/studyResult/8539.

[18] Kellier‐Steele N , Gilsenan AW , Midkiff K , et al. Assessing the incidence of a rare cancer utilizing cancer registry linkage to claims data in the U.S.: an update on two complementary ongoing safety studies. [Presented at the International Conference on Pharmacoepidemiology and Therapeutic Risk Management; August 2017. Montréal, Canada].

[19] US Census Bureau . Annual estimates of the resident population by age and sex for the United States and states: 2003–2016. Washington, DC: US Census Bureau; 2017.

