# Supplementary material for: Teriparatide Did Not Increase Adult Osteosarcoma Incidence in a 15‐Year US Postmarketing Surveillance Study
Source: J Bone Miner Res. 2020 Oct 13;36(2):244–51. doi: 10.1002/jbmr.4188 (PMC7988650; doi:10.1002/jbmr.4188)
Supplement: Supplementary file 1 — Supplemental Appendix Results for Patients With Other Diagnostic Codes [file JBMR-36-244-s001.docx]

# Supplemental Appendix: Results for Patients With Other Diagnostic Codes

Although not part of the primary study objective, patients diagnosed with one of five additional *International Classification of Diseases for Oncology, Third Edition* (ICD-O-3) morphology codes for which the primary tumor site was bone were also interviewed and identified cases were included in a sensitivity analysis. As of December 31, 2018, participating cancer registries had identified 1,727 patients; 1,193 patients were reported to the study center with contact information and met all necessary requirements to allow the study center to contact them for an interview, and 557 (32%) of these patients were interviewed.

## Patient Characteristics

Among patients diagnosed with one of five additional ICD-O-3 morphology codes, most were white (89%), and slightly more than half were men (53%). The mean age at the time of diagnosis was 65 years (range, 41-100 years). Two-thirds (374 of 557) were living at the time they were reported. The distribution of the five additional ICD-O-3 morphology codes for the 557 interviewed patients was dedifferentiated chondrosarcoma (n = 217, 39%); sarcoma, not otherwise specified (n = 135, 24%); malignant fibrous histiocytoma (n = 100, 18%); spindle cell sarcoma (n = 60, 11%); and fibrosarcoma, not otherwise specified (n = 45, 8%).

As with the osteosarcoma cases, although the primary anatomical site of the tumor varied, the tumors occurred primarily in the lower extremities. The most common site was the leg bones (n = 239, 43%), followed by the pelvic region (n = 107, 19%) and the upper limbs (n = 90, 16%). Other sites included the ribs, sternum, or clavicle (n = 37, 7%); vertebrae (n = 33, 6%); skull, face, or mandible (n = 31, 6%); and bone and joints, unspecified (n = 20, 4%).

## Medication Exposure

Of the 557 patients with one of the five additional ICD-O-3 codes who were interviewed, 1 patient reported a prior history of teriparatide treatment, which was confirmed. An additional 13 patients diagnosed in 2017 were interviewed; none reported prior exposure to teriparatide.

## Generalizability

For patients diagnosed with one of the five additional ICD-O-3 codes, no differences were notable in the distributions for age at diagnosis, sex, and ethnicity between all patients identified by participating registries and patients interviewed, although white patients represented a higher proportion of patients interviewed (89%) than of all patients identified (80%). The same trends for completion of interviews based on vital status were seen in this group as were seen for patients diagnosed with osteosarcoma. In addition, the distributions of ICD-O-3 morphology codes and primary tumor sites were also similar between identified and interviewed patients.
